# Current Landscape of Molecular Diagnostic Tests and Emerging Tools for Tuberculosis and Drug Resistance

**DOI:** 10.3390/diagnostics16132133

**Published:** 2026-07-07

**Authors:** Safaa El Kassimi, Rkia Eddabra, Bouchra Belkadi, Abdelkarim Filali-Maltouf, Hassan Ait Benhassou

**Affiliations:** 1 Laboratory of Microbiology and Molecular Biology, Faculty of Sciences of Rabat, Mohammed V University, Rabat 10100, Morocco; s.elkassimi@mascir.ma (S.E.K.);; 2 Prevention & Therapeutics Center, Moroccan Foundation for Advanced Science, Innovation and Research (MAScIR), Mohammed VI Polytechnic University (UM6P), Ben Guerir 43150, Morocco; 3 Higher Institute of Nursing Professions and Technical Health, Agadir 80000, Morocco

**Keywords:** Tuberculosis (TB), *Mycobacterium tuberculosis*, molecular diagnostics, nucleic acid amplification tests (NAATS)

## Abstract

This review synthesizes recent advances in WHO-endorsed NAATs for TB diagnosis and drug resistance detection. We examine the principles, genetic targets, diagnostic performance, implementation settings, and programmatic role of assays across the spectrum of technological complexity, from cartridge-based platforms to line probe assays and sequencing-based technologies. In addition, we highlight emerging molecular tools that show promise for future WHO endorsement and may further strengthen decentralized and near-patient testing. Low- and moderate-complexity assays such as Xpert^®^ MTB/RIF, Xpert^®^ Ultra, and Truenat^TM^ MTB/MTB Plus have emerged as essential frontline tools for TB diagnosis and rifampicin resistance detection, especially in decentralized settings. LPAs, including GenoType^®^ MTBDR*plus* and MTBDR*sl*, extend resistance profiling to isoniazid, fluoroquinolones, and second-line injectables, and remain valuable in intermediate and central laboratories. More recent developments, including Xpert^®^ XDR, Deeplex^®^ Myc-TB, AmPORE-TB^®^, and TBseq^®^, enable broader resistance detection and, in the case of targeted sequencing assays, comprehensive characterization of multidrug-resistant and extensively drug-resistant TB (MDR/XDR-TB). Emerging diagnostic innovations—such as CRISPR-based detection systems, streamlined isothermal amplification assays, and portable sequencing technologies—further expand the landscape and may complement existing WHO-endorsed platforms. Importantly, these technologies reduce delays in regimen selection, improve patient outcomes, and provide critical data for surveillance. Nevertheless, performance gaps for rare mutations, limited sensitivity in paucibacillary or extrapulmonary disease, infrastructure requirements, and cost remain barriers to universal adoption. The evolution of TB molecular diagnostics demonstrates a clear shift toward more rapid, accurate, and comprehensive resistance detection. No single assay is universally optimal, yet the combined portfolio, spanning rapid cartridge-based NAATs, LPAs, and next-generation sequencing, forms a complementary framework for improving diagnosis, optimizing treatment, and supporting global TB elimination strategies.

## 1. Introduction

Tuberculosis (TB) is one of the most prevalent infectious diseases worldwide, consistently ranking among the leading causes of illness and death despite sustained control efforts. In 2023, the World Health Organization (WHO) estimated that 10.8 million people developed TB, resulting in 1.25 million deaths, with an additional 161,000 deaths among individuals living with HIV [1]. More than 95% of TB-related deaths occur in low- and middle-income countries, where fragile laboratory infrastructure, delays in diagnosis, and limited access to molecular testing fuel ongoing transmission [2,3,4]. While pulmonary TB constitutes most cases and drives person-to-person spread, extrapulmonary forms represent a substantial share of the global burden, especially in immunocompromised populations, where they may account for up to 60% of cases [5,6].

The cornerstone of TB control is timely and accurate diagnosis, yet conventional methods remain inadequate. Sputum smear microscopy, long valued for its simplicity and low cost, has poor sensitivity, particularly among children, people living with HIV, and patients with paucibacillary disease [7]. Moreover, it cannot distinguish members of the *Mycobacterium tuberculosis* complex (MTBC) from non-tuberculous mycobacteria (NTM). Culture, the historical gold standard, offers higher sensitivity and enables phenotypic drug susceptibility testing (DST), but the method is slow, often requiring weeks, and demands specialized biosafety infrastructure and highly trained staff [8].

Over the past two decades, nucleic acid amplification tests (NAATs) have reshaped the diagnostic landscape. By detecting MTBC DNA directly in clinical specimens and increasingly by identifying resistance-associated mutations, NAATs provide major advantages over microscopy and culture. They deliver higher sensitivity and specificity while reducing diagnostic turnaround time to just a few hours [9]. Several platforms have been endorsed by the WHO, including Xpert^®^ MTB/RIF and its successor Xpert^®^ MTB/RIF Ultra (Xpert^®^ Ultra, Cepheid, Sunnyvale, CA, USA), both of which can confirm TB and detect rifampicin (RIF) resistance in under two hours [10,11].

NAATs are broadly categorized into two groups [12]. The first includes initial diagnostic tests, applied to clinical specimens from presumptive TB patients, often at the first point of care. The second comprises follow-on tests, used for bacteriologically confirmed TB cases to detect genetic markers of drug resistance. Within these groups, assays are further stratified by operational complexity. Low-complexity tests such as Xpert^®^ MTB/RIF, Xpert^®^ Ultra, Truenat^TM^ MTB (Molbio Diagnostics, Goa, India), and TB-LAMP (Eiken Chemical Company Ltd., Tokyo, Japan) are designed for peripheral or point-of-care settings, where infrastructure is minimal [13,14,15]. Moderate-complexity platforms, including Abbott RealTime MTB (Abbott Molecular, Chicago, IL, USA; hereinafter RT-MTB), BD MAX (Becton Dickinson, Franklin Lakes, NJ, USA), cobas^®^ MTB (Roche Diagnostics, Basel, Switzerland), and FluoroType^®^ MTB (Bruker, Bremen, Germany), require laboratory facilities and trained personnel, making them suitable for district or regional laboratories [16]. At the highest tier, centralized reference laboratories deploy complex assays such as Deeplex^®^ Myc-TB (Genoscreen, Lille, France and Illumina, San Diego, CA, USA), AmPORE-TB^®^ (Oxford Nanopore Diagnostics, Oxford, United Kingdom), and TBseq^®^ (Shengting Medical Technology Co., Ltd., Tianjin, China), which provide expanded drug-resistance profiling but demand advanced infrastructure, specialized expertise, and rigorous quality assurance [17,18].

Although NAATs perform well in pulmonary TB, including smear-negative cases, their role in extrapulmonary TB is more variable, reflecting differences in specimen type and bacillary load. Nonetheless, growing evidence supports their use in cerebrospinal fluid, pleural fluid, and lymph node aspirates, where they can substantially reduce diagnostic delay, even if a negative result cannot fully exclude disease [5,7,11].

Today, the diagnostic pipeline for TB is expanding at an unprecedented pace. More than 50 molecular assays are in advanced stages of development or evaluation, many aiming to close persistent gaps in sensitivity, drug-resistance detection, and operational feasibility [12,13]. Beyond WHO-endorsed platforms, a new generation of emerging molecular tools, including CRISPR-based detection systems, novel isothermal amplification assays, and portable sequencing technologies is rapidly advancing [19,20,21,22]. These innovations have the potential to complement established NAATs by improving decentralization, reducing time-to-result, and expanding access to resistance detection in resource-limited settings. Evidence from recent studies highlights the high diagnostic accuracy of CRISPR-based detection for TB [19,20], advances in isothermal amplification methods suitable for field deployment [22], and the increasing utility of portable nanopore sequencing for rapid drug-resistance profiling [21].

These innovations are deeply aligned with the vision of the WHO End TB Strategy, which identifies universal access to rapid molecular testing as a cornerstone of effective TB control [2]. Against this backdrop, this review seeks to provide a comprehensive synthesis of WHO-endorsed NAATs for TB diagnosis and drug-resistance detection. In addition, we outline key emerging diagnostic tools that are currently under evaluation and may shape the next generation of decentralized TB testing. We highlight not only their diagnostic performance but also their operational requirements and programmatic implementation, offering an integrated perspective on how these tools can be most effectively deployed within diverse health systems.

## 2. I-First-Line Diagnostic Assays for the Diagnosis of TB

### 2.1. Xpert^®^ MTB/RIF Assay

The introduction of the Xpert^®^ MTB/RIF (Cepheid, Sunnyvale, CA, USA) assay marked a watershed moment in the field of TB diagnostics. As the first fully automated, cartridge-based NAAT, it fundamentally reshaped diagnostic practices by enabling rapid and simultaneous detection of the MTBC and rifampicin resistance directly from patient specimens. Endorsed by the WHO in 2010, the assay represented a paradigm shift from laborious culture-based methods, often requiring weeks of incubation and specialized facilities, to a streamlined approach in which sample preparation, amplification, and real-time detection were consolidated within a single, closed cartridge system. This design not only minimized biosafety concerns but also significantly reduced the risk of cross-contamination [9].

The assay is based on real-time polymerase chain reaction (RT-PCR) technology and targets the 81-base pair rifampicin resistance-determining region (RRDR) of the *rpoB* gene. The cartridge contains lyophilized reagents, and the workflow is straightforward; after the addition of a sample reagent to liquefy and inactivate sputum, the mixture is transferred into the cartridge, which is then inserted into the GeneXpert instrument (Cepheid, Sunnyvale, CA, USA). Results for both TB detection and rifampicin resistance are available in under two hours, making it substantially faster than culture-based methods [9,23].

Large-scale multi-country evaluations confirmed its diagnostic impact. Pooled sensitivity against culture reached approximately 89% overall, exceeding 95% in smear-positive disease and around 67% in smear-negative cases, while specificity remained consistently above 98% [19,20]. Rifampicin resistance detection was similarly robust, with sensitivity and specificity greater than 95% compared with phenotypic drug susceptibility testing (DST) [9,21]. Based on this evidence, WHO initially recommended the assay for patients at risk of multidrug-resistant TB (MDR-TB) or TB/HIV co-infection, and by 2013 expanded its use as the preferred initial test for all persons with presumptive pulmonary TB [1,23].

From an operational perspective, Xpert was designed with decentralized deployment in mind. Instruments are available in various configurations, from single-module units suitable for peripheral laboratories to 80-module systems for high-throughput settings, with the four-module system being the most widely adopted. The closed cartridge format requires minimal hands-on time and limited technical expertise. However, the system is not without challenges: the high cost of instruments and cartridges, reliance on stable electricity, and need for annual calibration have posed barriers in resource-limited environments, even under concessional pricing schemes brokered by global health partners [23,24].

Beyond pulmonary TB, Xpert has been tested extensively for extrapulmonary specimens, including cerebrospinal fluid, pleural fluid, lymph node aspirates, and tissue biopsies, where sensitivity varies by sample type and bacillary burden. While a negative result does not rule out disease, especially in extrapulmonary TB, the assay has nonetheless shortened diagnostic delays and facilitated earlier treatment initiation in many high-burden countries [5,6,25].

The programmatic impact of Xpert^®^ MTB/RIF has been profound. Widespread roll-out improved case detection accelerated initiation of effective therapy and provided valuable surveillance data on rifampicin resistance. Modeling analyses suggest that its deployment reduced diagnostic delays and may have averted hundreds of thousands of deaths globally [11,23]. Yet limitations remain true point-of-care access is still restricted, sample transport can delay testing, and diagnostic capacity is not always matched by linkage to treatment. These gaps highlighted the need for further innovation and directly informed the development of the Xpert^®^ Ultra (Cepheid, Sunnyvale, CA, USA), which was designed to improve sensitivity in paucibacillary disease, pediatric populations, and extrapulmonary forms of TB [10].

### 2.2. Xpert^®^ MTB/RIF Ultra (Xpert^®^ Ultra)

Introduced in 2017, Xpert^®^ Ultra represents the second-generation cartridge-based NAAT within the GeneXpert^®^ platform, developed to address the limitations of the original Xpert assay in detecting paucibacillary TB. WHO rapidly endorsed Xpert^®^ Ultra in the same year as the preferred initial diagnostic test for all individuals with presumptive TB, recommending its replacement of the first-generation assay in most programmatic settings [25].

Like its predecessor, Ultra is performed on the GeneXpert platform and integrates specimen processing, DNA extraction, real-time PCR amplification, and detection within a single-use cartridge. However, several technical innovations distinguish Xpert Ultra. Beyond retaining the *rpoB* target for rifampicin resistance detection, the assay amplifies two multicopy insertion elements (IS6110 and IS1081), thereby increasing template abundance. Combined with a larger reaction chamber (50 µL vs. 25 µL) and improved cartridge fluidics, this dual-target strategy reduces the limit of detection from approximately 131 CFU/mL with Xpert MTB/RIF to just 16 CFU/mL [11,26].

Another major innovation is the introduction of the “trace” category, which signals the presence of TB DNA at concentrations below the lowest semi-quantitative range of the original assay. This feature significantly improves detection in children, people living with HIV (PLHIV), and patients with extrapulmonary or meningeal TB. Nonetheless, a trade-off exists: specificity may be slightly reduced in patients with a history of TB, where residual non-viable DNA can lead to false-positive results. Accordingly, WHO advises cautious interpretation of trace calls in previously treated individuals and recommends clinical or laboratory confirmation where feasible [27,28].

Head-to-head multicountry evaluations have confirmed Ultra’s superior sensitivity. Among smear-negative, culture-positive patients, sensitivity increased from 67% with Xpert MTB/RIF to 78% with Xpert Ultra, while specificity remained above 96% [29]. In HIV-positive cohorts, Xpert Ultra detected 13–17% more culture-confirmed cases [30], and in children, sensitivity improved to 74% compared to 62% with the first-generation assay [31]. Notably, in TB meningitis, sensitivity rose to 95% versus 77% with Xpert MTB/RIF in prospective studies [32]. For rifampicin resistance, Xpert Ultra maintains accuracy comparable to its predecessor, with pooled sensitivity and specificity of around 95% and 98%, respectively, compared with phenotypic DST [33,34]. Importantly, Xpert Ultra also reduces indeterminate results in low-burden specimens, providing more actionable outcomes [35].

Programmatic data confirm these laboratory findings. Implementation studies from Uganda, South Africa, and India demonstrated increases of 5–15% in bacteriologically confirmed TB notifications after Xpert Ultra rollout, with the largest gains in pediatric and HIV care settings [34,36]. Operationally, the transition was seamless since the same GeneXpert instruments are used, with equivalent cartridge pricing (US$9.98) and similar turnaround time of two hours [37]. Yet persistent challenges, such as dependence on electricity, annual calibration, and fragile supply chains, continue to hinder equitable access in many high-burden regions [10].

### 2.3. Truenat MTB, MTB Plus, and Truenat RIF Dx^®^

The Truenat MTB, MTB Plus, and Truenat RIF Dx assays, developed by Molbio Diagnostics (formerly BigTec Labs, Bengaluru, India), represent a family of chip-based, battery-operable, real-time micro-PCR tests specifically designed for decentralized TB diagnosis. First introduced in India in 2013, these assays have progressively evolved to address diagnostic gaps in peripheral and primary health-care settings. In 2020, the WHO endorsed Truenat MTB as an initial diagnostic test for TB and Truenat RIF Dx as a follow-on test for rifampicin resistance, while the MTB Plus variant, launched in 2019, expanded genetic coverage to further improve sensitivity and reduce the risk of false negatives arising from polymorphisms in target regions [38,39].

Importantly, WHO classifies Truenat as a low-complexity automated NAAT, positioning it as a practical solution for resource-constrained environments. Its modular, portable design and minimal infrastructure requirements enable near point-of-care deployment, bridging the gap between community-level facilities and centralized molecular platforms that remain inaccessible in many high-burden countries [38].

The Truenat workflow is deliberately simple. It comprises two compact instruments: the Trueprep Auto (Molbio Diagnostics Pvt. Ltd., Bengaluru, India), which conducts automated nucleic acid extraction with magnetic bead purification in under 20 min, and the Truelab analyser (Molbio Diagnostics Pvt. Ltd., Bengaluru, India), a lightweight real-time PCR device. Once DNA is extracted, the eluate is transferred to a disposable micro-PCR chip containing lyophilised reagents. The Truelab analyser, available in UnoDx (one module), Duo (two modules), and Quattro (four modules) configurations, then performs amplification and detection within 35–50 min. Notably, the fully enclosed system minimizes contamination risk while requiring very little hands-on time, an essential advantage in peripheral laboratories [40].

At the molecular level, Truenat MTB and MTB Plus target the detection of *Mycobacterium tuberculosis* complex DNA, while Truenat RIF Dx interrogates the rifampicin resistance-determining region (RRDR) of the *rpoB* gene, aligning with the targets of other WHO-endorsed NAATs [41]. The assays employ TaqMan^®^ probe chemistry within a chip-based thermocycler, allowing precise thermal control and rapid cycling despite the compact form factor [42].

Diagnostic performance has been consistently robust. In a large multicentre trial coordinated by the Indian Council of Medical Research (ICMR) across 100 sites, Truenat MTB achieved sensitivity and specificity of 89% and 99% against culture, while MTB Plus improved sensitivity to 92% in smear-negative, culture-positive disease [39]. For rifampicin resistance detection, RIF Dx demonstrated sensitivity and specificity of 97% and 99% compared with phenotypic DST and LPA assays [43]. WHO meta-analyses further concluded that Truenat assays offer diagnostic accuracy broadly comparable to Xpert MTB/RIF, with the added advantage of decentralised placement [38].

Notably, Truenat has shown particular value in high-risk groups. Among people living with HIV, MTB Plus achieved sensitivity of 86% for smear-negative TB, outperforming smear microscopy and approaching the performance of Xpert Ultra [44]. Moreover, in extrapulmonary specimens such as cerebrospinal fluid and lymph node aspirates, Truenat markedly outperformed smear and culture, underscoring its utility where bacillary load is often low [45,46].

India’s National TB Elimination Programme has scaled Truenat across more than 10,000 primary health centres and district hospitals [47]. Field evaluations, such as those in Odisha, revealed that on-site Truenat testing reduced the median time to treatment initiation from six days (with centralized Xpert testing) to just one day, significantly decreasing pre-treatment loss to follow-up [48]. These programmatic outcomes illustrate how decentralised molecular testing can accelerate patient management and improve retention in care.

Finally, cost-effectiveness analyses reinforce Truenat’s role in high-burden settings. While individual test costs (US$13–15 for MTB/Plus; US$15–17 for RIF Dx) exceed those of smear microscopy, the reduction in diagnostic delays and transmission renders the platform highly cost-effective [49]. Truenat instruments are less expensive than GeneXpert systems and require fewer infrastructure investments, making them particularly attractive for national TB programs [50].

### 2.4. TB-LAMP

The TB-LAMP assay, developed by Eiken Chemical Co. (Tokyo, Japan), represents one of the earliest attempts to provide a low-complexity molecular alternative to smear microscopy for the detection of MTBC DNA in sputum. Endorsed by the WHO in 2016, it was recommended as a replacement for smear microscopy in adults presenting with pulmonary TB symptoms. Importantly, TB-LAMP embodies the concept of decentralised NAAT, offering rapid and robust results in peripheral laboratories where access to conventional PCR platforms remains limited [15].

Unlike standard PCR, which relies on repeated thermal cycling, TB-LAMP uses loop-mediated isothermal amplification (LAMP), a technique first described by Notomi et al. in 2000. This method operates at a constant temperature of approximately 65 °C, exploiting the strand displacement activity of *Bst* polymerase and multiple primers that target distinct regions of the genome, including loop primers that accelerate amplification. Notably, the Eiken assay focuses on the IS6110 insertion sequence, present in multiple copies across the MTBC genome, thereby enhancing analytical sensitivity [15,51,52]. Amplification is completed within 40–60 min, with results interpreted visually under UV light as a bright green fluorescence signal, eliminating the need for sophisticated readout devices [15,53].

The workflow was deliberately designed for simplicity. DNA can be released through a crude lysis step or purified using the Loopamp PURE kit (Eiken Chemical Co., Ltd., Tokyo, Japan), then directly added to lyophilised reaction tubes. Amplification occurs in the compact LF-160 device (Eiken Chemical Co., Ltd., Tokyo, Japan), a portable heating block that maintains isothermal conditions. Moreover, the visual calcein–manganese detection system further reduces equipment needs, though training may be required to avoid misinterpretation of weak signals. This minimalistic yet functional setup has proven particularly attractive for laboratories with unstable electricity supply and limited biosafety infrastructure [15,54,55,56,57,58].

Diagnostic performance has been evaluated extensively. A large multicountry study coordinated by WHO and FIND, spanning 14 countries and more than 4600 participants, reported pooled sensitivity of around 81% and specificity of 96% compared with culture in smear-positive TB. Although sensitivity in smear-negative, culture-positive disease dropped to 46–50%, TB-LAMP consistently outperformed smear microscopy across all clinical groups [15,54,55]. In HIV-positive patients, sensitivity declined modestly, especially with advanced immunosuppression, but specificity remained consistently high [56]. These findings underscore TB-LAMP’s clinical value, particularly in contexts where smear microscopy remains the primary diagnostic tool.

From an implementation perspective, TB-LAMP offers several operational advantages. Reagents are stable without a cold chain, the portable device functions in resource-constrained environments, and the assay shows tolerance to some sputum inhibitors. In addition, its affordability, estimated at US$6–8 per test positions it as a pragmatic option in regions where Xpert coverage is incomplete [51,59,60]. Importantly, WHO guidance highlights TB-LAMP as a replacement for smear microscopy or as a follow-on test where Xpert is unavailable. Nevertheless, its utility is constrained by the absence of drug resistance detection and its lack of validation for extrapulmonary samples, which limit its role in settings with high MDR-TB burden [15].

Despite WHO endorsement, global uptake has been modest. This is largely due to the rapid expansion of Xpert MTB/RIF and Xpert Ultra, which combine higher sensitivity with simultaneous rifampicin resistance detection. Yet, TB-LAMP remains relevant in contexts where GeneXpert access is constrained, or cartridge supply chains are unreliable. Indeed, countries such as Cambodia and Cameroon have incorporated TB-LAMP into national diagnostic algorithms, often reserving GeneXpert for smear-negative cases or drug resistance screening [37].

Finally, research is actively exploring the next generation of LAMP-based TB diagnostics. Multiplex formats, incorporation of resistance-associated targets, and integration into microfluidic devices are under development, aiming to expand diagnostic scope and enable near-patient testing. These innovations suggest that, while TB-LAMP may serve as an interim solution today, it could also provide the foundation for more sophisticated, portable molecular platforms of the future [61].

### 2.5. Abbott RealTime MTB and MTB RIF/INH

The Abbott RealTime MTB assay is a fully automated real-time polymerase chain reaction (PCR) test developed by Abbott Molecular (Illinois, USA) for qualitative detection of MTBC DNA. Commercially launched in 2013, it runs on the Abbott m2000 platform, a high-throughput molecular system already widely used for HIV and hepatitis viral load monitoring [62,63]. Importantly, In 2021, the World Health Organization included the Abbott RealTime MTB assay among the moderate-complexity automated nucleic acid amplification tests (NAATs) recommended for the diagnosis of pulmonary tuberculosis, indicating its suitability for use in moderate- to high-complexity laboratory settings as part of the WHO policy framework for rapid molecular diagnostics [64].

The m2000 platform integrates two modules: the m2000sp, which automates nucleic acid extraction through magnetic bead technology, and the m2000rt, which performs real-time PCR amplification and detection using dual-labeled hydrolysis probes (TaqMan chemistry) [65]. Notably, RealTime MTB targets both the IS6110 insertion sequence and the *pab* gene, offering redundancy in detection and thus maintaining accuracy in strains with few or absent IS6110 copies, a feature of particular value in some Asian regions [66,67]. In fact, the dual-target strategy not only enhances sensitivity but also minimizes the risk of false negatives caused by genomic variability. Manufacturer data report a limit of detection as low as 17 CFU/mL for MTB. Moreover, the complementary RealTime MTB RIF/INH Resistance assay, with a detection limit of 60 CFU/mL, interrogates *rpoB* (rifampicin resistance), *katG*, and the *inhA* promoter (isoniazid resistance), using the same DNA extract. This enables same-day confirmation of both TB diagnosis and resistance profiles [68,69]. Reflecting this value, WHO endorsed the combined MTB and RIF/INH workflow in 2017 for deployment in well-equipped laboratories [64].

Clinical performance evaluations underscore its accuracy. For example, in South Africa, RealTime MTB achieved a sensitivity of 92.7% and a specificity of 99.2% compared with culture, notably outperforming Xpert MTB/RIF in smear-negative, culture-positive cases [70]. Clinical performance evaluations consistently report sensitivities above 90% and specificities approaching 99%, with particularly strong performance in people living with HIV [71,72]. Importantly, the dual-target design enhances robustness against IS6110-low strains, which have historically challenged other NAATs [67].

From an operational perspective, the assay is best suited to centralized or reference laboratories due to its infrastructure needs, high-throughput capacity, and instrument cost. The m2000 system can process hundreds of samples daily, making it highly attractive for high-burden programs, especially when integrated into HIV laboratories already running Abbott platforms [73]. Programmatic data from Kenya and Uganda illustrate that co-utilization for HIV viral load monitoring and TB diagnostics improves laboratory efficiency and reduces per-test costs [74].

In terms of affordability, the assay is priced at approximately US$14–16 per MTB test and US$18–20 per RIF/INH test, although capital equipment costs exceed US$100,000 [75]. Nevertheless, in high-throughput contexts, economies of scale substantially lower operational costs, particularly when the system is multiplexed across multiple disease programs [74,76].

The assay implementation has been most common in national reference laboratories in Africa and Asia, and in low-incidence, high-resource countries where the assay’s high specificity supports clinical confidence [37]. Its strong performance in smear-negative and HIV-positive populations highlights its clinical value in high-burden settings, while its high specificity benefits low-burden regions where false positives carry significant public health implications [66,77,78].

### 2.6. BD MAX™ MDR-TB

The BD MAX™ MDR-TB Becton, Dickinson and Company (BD), Franklin Lakes, NJ, USA) assay represents a fully automated, cartridge-based NAAT designed to simultaneously detect MTBC and resistance to RIF and isoniazid (INH). Developed for use on the BD MAX platform, the assay has been classified by WHO as a moderate-complexity NAAT and is recommended for respiratory samples in individuals with presumptive pulmonary TB. Importantly, it offers a rapid alternative to culture and phenotypic drug susceptibility testing (DST) in laboratories with adequate infrastructure [79,80].

The BD MAX system integrates DNA extraction, amplification, detection, and automated interpretation in a single closed platform, with capacity for up to 24 samples per run and a turnaround time of less than four hours. However, while the system is often described as fully automated, the workflow involves two sequential steps: MTBC detection is performed first, and only specimens testing MTBC-positive are then manually transferred within the instrument workflow for rifampicin and isoniazid resistance determination. The analytical sensitivity is reported at 0.5 CFU/mL for MTBC detection and 6 CFU/mL for resistance determination [81]. Notably, the assay employs a multiplex real-time PCR strategy targeting IS6110, IS1081, and the single-copy gene *devR* (Rv3133c), thereby safeguarding sensitivity in strains with low IS6110 copy numbers [79]. Resistance detection focuses on canonical mutations within the rifampicin resistance-determining region (RRDR) of *rpoB* and the well-characterized *katG* 315 codon and *inhA* promoter mutations, enabling accurate classification of both MDR-TB and isoniazid-monoresistant TB [82]. In addition, the assay provides a semi-quantitative interpretation category designated as “MTB low positive,” which reflects detection of M. tuberculosis DNA near the limit of assay sensitivity; in such cases, resistance calls may be indeterminate and repeat testing or correlation with clinical and microbiological findings is recommended for accurate interpretation.

Importantly, clinical performance has been confirmed across multicountry and single-centre studies. A pivotal evaluation reported pooled sensitivity and specificity above 90% for MTBC detection, with high concordance for RIF/INH resistance when compared with phenotypic DST and sequencing [83]. Comparative analyses of centralized molecular platforms, including Abbott RealTime MTB, Hain FluoroType, Roche cobas, and BD MAX, showed that BD MAX performs robustly across resistance targets [84]. Moreover, real-world data from Korea highlighted its clinical impact, demonstrating 79.5% sensitivity for pulmonary TB detection, 100% sensitivity and specificity for INH resistance, and meaningful reductions in the time to treatment initiation from a median of 5.5 days to just two days [85]. In bronchoscopy-derived samples, the assay outperformed smear microscopy, achieving nearly 80% sensitivity and close to 89% specificity [86].

Otherwise, BD MAX offers several advantages for regional or reference laboratories. Ease-of-use evaluations underscore its reduced hands-on time compared with other moderate-complexity NAATs, an important feature for high-throughput programs [37]. The ability to detect isoniazid resistance alongside rifampicin resistance is particularly valuable, as isoniazid-resistant TB is more common than MDR/RR-TB in many settings and requires distinct treatment regimens [87].

Nevertheless, the assay is not without limitations. As with other molecular tests, its scope is confined to targeted loci, and resistance mutations outside these regions may go undetected. For example, rare *rpoB* mutations beyond the RRDR or non-canonical *inhA* variants can produce false-susceptible results, making confirmatory phenotypic or sequencing approaches essential in discordant cases [82,88]. In addition, performance estimates for RIF resistance may be imprecise in cohorts with limited resistant isolates, emphasizing the need for multicenter validation [85,86].

WHO’s operational handbook also notes that BD MAX requires stable electricity, climate control, and strict molecular laboratory safeguards against contamination, restricting its deployment to centralized or regional facilities [80]. Yet in these environments, the assay provides same-day confirmation of TB and first-line resistance, significantly accelerating treatment decisions compared with culture-based DST [89].

Looking forward, emerging evidence suggests that BD MAX may extend its utility beyond sputum. Preliminary studies have reported encouraging results in extrapulmonary specimens, though further validation will be necessary before widespread policy adoption [90]. Ongoing research is also assessing its role in active case-finding programs and benchmarking its performance against newer-generation assays such as Xpert Ultra and Abbott RealTime MTB [91].

### 2.7. FluoroType^®^ MTB

The FluoroType^®^ MTB assay (Bruker-Hain Lifescience, Nehren Germany) is a moderate-complexity molecular test developed for the direct detection of MTBC DNA from clinical specimens. It combines real-time PCR with the proprietary FluoroCycler^®^ platform and employs HyBeacon probes that emit specific fluorescence signals upon hybridization, thereby ensuring high analytical sensitivity and specificity while minimizing the risk of cross-contamination. Initially designed for pulmonary TB diagnosis, the assay later evolved into the FluoroType^®^ MTBDR version, which incorporates resistance detection to rifampicin and isoniazid through targeting the *rpoB*, *katG*, and *inhA* promoter regions. This extension enables rapid molecular DST compared with conventional phenotypic methods.

Importantly, the workflow is fully automated following DNA extraction, delivering results in roughly three hours. The platform can process up to 96 samples per run, making it most suitable for centralized or reference laboratories. Its closed-cartridge design substantially reduces the risk of amplicon contamination, a common limitation of conventional PCR workflows. The FluoroCycler^®^ instrument Hain Lifescience GmbH, Nehren, Germany integrates amplification, melting-curve analysis, and result interpretation within a single run. Nevertheless, sample preparation remains a critical determinant of accuracy; despite the availability of automated extraction options, performance depends heavily on DNA quality, especially in paucibacillary or extrapulmonary specimens [92,93].

Several independent evaluations highlight the assay’s diagnostic robustness. In smear-positive, culture-confirmed pulmonary TB, sensitivity reaches 95–98%, with specificity exceeding 98% across diverse settings [94,95]. For smear-negative disease, sensitivity falls to 60–75% but remains markedly higher than microscopy and broadly comparable with other WHO-recommended NAATs such as Xpert MTB/RIF. Studies from Europe and Africa further confirm the platform’s reproducibility, showing minimal operator variability and consistent results in programmatic use [37,96]. The MTBDR version has also shown strong concordance with phenotypic DST, with pooled sensitivity and specificity for rifampicin resistance around 92–96% and 98–99%, respectively, and for isoniazid resistance approximately 85–90% and 98% [97,98]. However, as with other targeted NAATs, rare mutations outside canonical regions may escape detection, leading to occasional discordance with whole-genome sequencing.

From an implementation perspective, FluoroType^®^ MTB/MTBDR is well suited for medium- to high-throughput laboratories. Although it is not intended as a true point-of-care assay, its scalability and compatibility with existing laboratory infrastructure render it attractive for national reference centers. Compared with Xpert, it requires more laboratory capacity and skilled staff, yet its larger batch size and lower per-sample costs in high-volume contexts offer a compelling trade-off [10]. Cost-effectiveness analyses indicate that integration into centralized diagnostic networks may reduce expenditure, particularly when the FluoroCycler^®^ platform is also applied to other infectious disease testing [99].

Programmatically, the assay’s main strengths lie in its throughput, robust contamination control, and reliable detection of rifampicin and isoniazid resistance. Its key limitations include reduced sensitivity in smear-negative or extrapulmonary TB, reliance on high-quality DNA extraction, and restricted coverage of resistance mutations beyond the *rpoB*, *katG*, and *inhA* promoter loci. Moreover, compared with widely endorsed assays such as Xpert Ultra and Truenat, the absence of WHO endorsement continues to limit global uptake despite favorable evaluations. Nevertheless, head-to-head studies with sequencing-based methods and expansion of resistance coverage could clarify its place in future TB diagnostic strategies [18,89].

### 2.8. cobas^®^ MTB and cobas MTB-RIF/INH^®^

The cobas^®^ MTB and cobas MTB-RIF/INH assays (Roche Molecular Systems, Pleasanton, CA, USA) are fully automated, moderate-complexity NAATs designed for detection of MTBC and for simultaneous identification of RIF and INH resistance. Introduced in 2017 and subsequently endorsed by WHO in 2021 as part of the moderate-complexity NAAT class [39,99], these assays are intended for use on respiratory specimens from adults and children, including people living with HIV, and are particularly suited for centralized or high-throughput laboratories within tiered diagnostic networks.

Both assays run exclusively on the Roche cobas 6800/8800 platforms, which integrate chemical inactivation, magnetic-bead based nucleic acid extraction, multiplex real-time PCR, fluorescence-based detection, and automated result interpretation. The MTB assay targets two distinct genomic regions of MTBC to ensure lineage-independent detection, while the MTB-RIF/INH assay simultaneously interrogates the *rpoB* rifampicin resistance-determining region (RRDR), the *katG* codon 315, and the *inhA* promoter. Importantly, this design allows differentiation between MDR-TB (resistant to both RIF and INH) and isoniazid-monoresistant TB (Hr-TB), which requires adapted first-line regimens [100].

From an operational standpoint, the cobas system offers continuous random-access testing with full laboratory information system connectivity, enabling integration into multi-disease molecular diagnostic workflows. Notably, the same platforms support testing for HIV, hepatitis viruses, HPV, and SARS-CoV-2, which increases throughput and programmatic cost-efficiency. Analytical sensitivity is high, with reported limits of detection of 7.6 CFU/mL in sputum/BAL sediment and 8.8 CFU/mL in raw sputum for MTBC detection; for resistance detection, the LoD is 94 CFU/mL (RIF) and 12.6 CFU/mL (INH) in sediment, and 182 CFU/mL (RIF) and 27.5 CFU/mL (INH) in raw sputum [101,102]. Time to first MTB result is approximately three hours, with additional results every 90 min in continuous-run mode, while the cobas 8800 can deliver over 900 results within a standard 8-h shift [103].

Clinical evaluations consistently demonstrate high diagnostic accuracy. A multicentre study across Europe and Africa reported overall sensitivity of 92% compared with culture, with 100% sensitivity in smear-positive and around 85% in smear-negative cases, while specificity exceeded 99% [104]. For resistance detection, pooled analyses indicate sensitivities of 92–96% for RIF and 90–93% for INH, with specificities above 98% against phenotypic DST and sequencing [105,106]. Nevertheless, as emphasized by WHO evidence reviews, performance declines in paucibacillary specimens, a limitation shared with other mutation-targeted assays [39,107].

In practice, cobas MTB-RIF/INH performed on the same extract shortens time to treatment by avoiding referral for phenotypic DST, thereby reducing delays in high-burden settings [108]. Moreover, the scalability and random-access design of the cobas system make it attractive for regional laboratories managing diverse diagnostic workloads. Cost-effectiveness analyses suggest that while the per-test cost (US$16–18 for MTB and US$20–22 for MTB-RIF/INH) is higher than smear microscopy or LAMP assays, economies of scale and multi-disease testing contribute to overall savings in centralized laboratories [109,110].

Nevertheless, implementation requires reliable electricity, climate control, skilled molecular staff, and strong quality management systems. At present, WHO does not recommend the cobas assays for extrapulmonary or non-respiratory samples pending further validation. Finally, as with other targeted assays, rare resistance mutations outside the *rpoB*, *katG*, and *inhA* loci may yield false-susceptible results, underscoring the importance of confirmatory testing in discordant cases [39,111].

**Figure 1:**
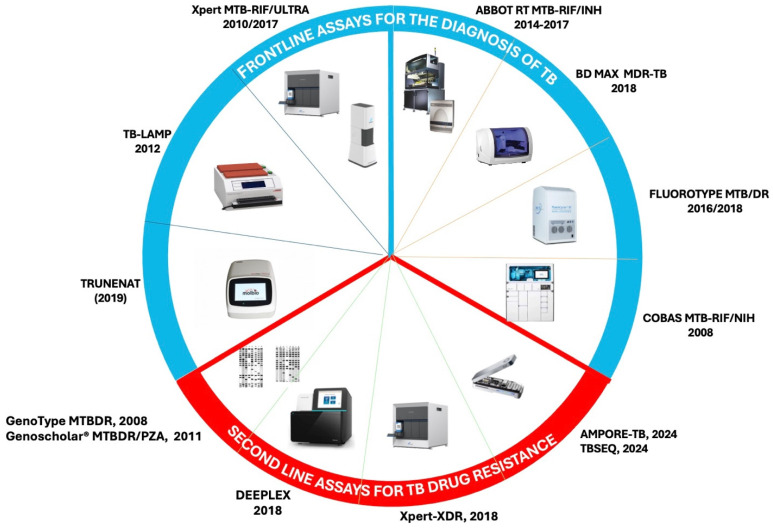
Classification of WHO-endorsed nucleic acid amplification tests (NAATs) for tuberculosis (TB) by clinical use. The blue segment shows frontline assays for initial TB diagnosis and rifampicin resistance detection (Xpert MTB/RIF, Xpert Ultra, Abbott RealTime MTB-RIF/INH, BD MAX MDR-TB, FluoroType MTB/DR, Cobas MTB-RIF/NIH, TB-LAMP, Truenat). The red segment shows second-line assays for extended drug resistance testing (GenoType MTBDR, Genoscholar MTBDR/PZA, Deeplex, Xpert XDR, AMPORE-TB, TBSEQ). Years indicate the products launch.

An overview of WHO-endorsed and commercial frontline assays for the diagnosis of tuberculosis, including their analytical characteristics, recommendations, benefits, and limitations, is summarized in Table 1. Their chronological introduction and positioning within diagnostic algorithms are illustrated in Figure 1.

## 3. II-Follow-On Assays for the Detection of TB and Drugs Resistance

### 3.1. The Xpert^®^ MTB/XDR Assay

Xpert^®^ MTB/XDR is a cartridge-based, automated nucleic acid amplification test designed to rapidly characterize resistance patterns beyond rifampicin, thereby supporting the programmatic management of advanced drug-resistant tuberculosis in line with the current WHO resistance definitions. Under this classification, *pre-XDR TB* refers to MDR/RR-TB with additional resistance to any fluoroquinolone, while *XDR-TB* is defined as MDR/RR-TB with resistance to a fluoroquinolone and at least one additional Group A drug (bedaquiline or linezolid). Although Xpert^®^ XDR does not directly detect resistance to bedaquiline or linezolid, its ability to reliably identify fluoroquinolone resistance enables early recognition of pre-XDR TB and prioritization of patients for further confirmatory testing and regimen optimization [39,99].

The assay is intended for reflex testing following detection of rifampicin resistance by Xpert^®^ MTB/RIF or Xpert^®^ Ultra and can be performed on residual sample reagent-treated material or decontaminated specimens, without requiring repeat sample collection. This approach reduces diagnostic delays and operational complexity and is compatible with routine laboratory workflows. Residual treated specimens may be reflexed within defined processing windows, typically up to 24–72 h when stored at 2–8 °C, without clinically relevant loss of performance, allowing timely escalation of drug-resistance testing [112,113].

Xpert^®^ XDR employs real-time PCR combined with melt-curve analysis and interrogates a comprehensive set of resistance-associated genomic targets. Rifampicin resistance is detected through the *rpoB* gene, while isoniazid resistance detection spans *katG* and the *inhA* promoter region, complemented by additional loci including the *fabG1* and *ahpC* promoter regions. The inclusion of these supplementary targets improves sensitivity for isoniazid resistance detection compared with assays limited to *katG* and *inhA* alone, particularly in settings where alternative resistance mechanisms are prevalent. Fluoroquinolone resistance is assessed through mutations in *gyrA* and *gyrB*, and resistance to second-line injectable agents through *rrs* and the *eis* promoter region.

Clinical validation studies have demonstrated robust performance across resistance categories. Sensitivity for fluoroquinolone resistance detection is consistently high, generally exceeding 90% relative to phenotypic DST, with specificities above 98%, supporting reliable identification of pre-XDR TB. Sensitivity for isoniazid resistance is moderate to high and superior to earlier cartridge-based assays due to the expanded target set, while specificity remains high. Performance for second-line injectable resistance detection is similarly strong, although variability is observed depending on circulating mutation profiles and reference standards [114,115].

From an operational perspective, Xpert^®^ XDR runs on existing GeneXpert platforms, delivers results in under two hours, and requires minimal additional training or infrastructure. The cartridge cost is approximately US$14–16 per test, higher than first-line Xpert assays but substantially lower and faster than culture-based DST or sequencing when used as an initial resistance screen. Within WHO-recommended diagnostic algorithms, Xpert^®^ XDR occupies a critical intermediary role, enabling rapid stratification of MDR/RR-TB cases into drug-resistance categories aligned with contemporary treatment regimens and facilitating earlier initiation of appropriate therapy [116,117,118,119].

### 3.2. GenoType^®^ MTBDRplus and GenoType^®^ MTBDRsl

The GenoType^®^ MTBDRplus and MTBDRsl assays (Bruker, Nehren, Germany) remain among the most widely implemented molecular diagnostics endorsed by WHO for detecting drug resistance in MTBC. Both assays are based on multiplex PCR amplification followed by reverse hybridization to oligonucleotide probes immobilized on nitrocellulose strips, with subsequent enzymatic colorimetric detection. This workflow, though requiring moderate laboratory infrastructure, allows simultaneous identification of MTBC and key resistance mutations within one to two days, substantially faster than conventional culture-based drug susceptibility testing (DST) [92].

MTBDRplus is specifically designed to detect resistance to rifampicin and isoniazid, the two cornerstone drugs of first-line TB therapy. It targets the *rpoB* gene for rifampicin resistance, and both *katG* codon 315 and the *inhA* promoter region for isoniazid resistance. Importantly, multicentre evaluations and meta-analyses consistently demonstrate excellent accuracy, with pooled sensitivity and specificity of 96–98% and >99% for rifampicin resistance, respectively, and somewhat lower sensitivity (85–90%) for isoniazid resistance, largely reflecting the broader diversity of isoniazid resistance mutations [9,120,121]. The ability to identify both rifampicin and isoniazid resistance simultaneously makes MTBDRplus a cornerstone in the rapid confirmation of multidrug-resistant TB (MDR-TB).

Building on this foundation, MTBDRsl extends resistance detection to critical second-line drugs. It interrogates mutations in *gyrA* and *gyrB* for fluoroquinolone resistance, *rrs* for second-line injectable resistance (amikacin, kanamycin, capreomycin), and the *eis* promoter for kanamycin resistance, with newer versions also incorporating *inhA* promoter probes relevant to ethionamide cross-resistance. Reported sensitivity for fluoroquinolone resistance ranges from 85–90%, with specificity consistently above 95%. By contrast, sensitivity for second-line injectables is somewhat lower (70–80%), reflecting the presence of rare or region-specific mutations not covered by the probe design [122,123,124].

WHO guidelines currently recommend MTBDRplus as an initial test for rifampicin and isoniazid resistance in patients with pulmonary TB, and MTBDRsl for patients with rifampicin-resistant or MDR-TB to detect additional resistance to fluoroquinolones and injectables. Notably, their ability to identify pre-XDR early facilitates faster initiation of optimized regimens. Indeed, programmatic data show that their use can reduce the time to treatment initiation by more than two weeks compared to culture-based DST [99,125].

Operationally, LPAs require moderate infrastructure, including designated areas for DNA extraction, amplification, and hybridization to minimize contamination risks. While less automated and more labor-intensive than cartridge-based NAATs, they are best suited to centralized or intermediate-level laboratories with PCR capacity. Their performance is strongest with smear-positive sputum or cultured isolates, as sensitivity declines in paucibacillary samples such as pediatric or extrapulmonary TB [39].

Cost-effectiveness analyses suggest a per-test price of US$20–25, excluding infrastructure and personnel costs. Although more resource-intensive than cartridge-based platforms, LPAs remain highly valuable in high MDR-TB burden settings, where their capacity to provide rapid and comprehensive resistance profiles supports appropriate regimen design, limits transmission, and improves clinical outcomes [39,99].

### 3.3. Genoscholar™ NTM + MDRTB II and Genoscholar™ PZA-TB II

The Genoscholar™ NTM + MDRTB II and Genoscholar™ PZA-TB II LPAs, developed by Nipro (Osaka, Japan), is a high-complexity molecular test designed for the simultaneous differentiation of *Mycobacterium tuberculosis complex* (MTBC) from selected non-tuberculous mycobacteria (NTM) and for the detection of rifampicin (RIF) and isoniazid (INH) resistance mutations. WHO classifies it within the LPA category for first-line resistance testing alongside Hain assays and recommends its use primarily on smear-positive sputum or culture isolates, while discouraging routine application on smear-negative samples due to reduced sensitivity [39,99].

The assay relies on multiplex PCR with biotinylated primers followed by reverse hybridization of amplicons to nitrocellulose strips containing immobilized probes. These probes target wild-type and mutation sequences within *rpoB* (RIF resistance), *katG* codon 315, and the *inhA* promoter region, in addition to probes for MTBC and selected NTMs such as *M. avium*, *M. intracellulare*, and *M. kansasii*. Detection is achieved via streptavidin–alkaline phosphatase conjugation with chromogenic readout, interpreted visually or with dedicated strip readers. The workflow typically requires 5–8 h from DNA extraction to result, enabling same-day testing for smear-positive specimens and <24 h for culture isolates [126,127].

While analytical limits of detection (LoD) are not expressed in CFU/mL, practical performance indicates reliable use only in smear-positive sputum (bacillary loads ≥10^4^ CFU/mL) or cultured isolates. In multicentre evaluations led by FIND and WHO, NTM + MDRTB II demonstrated pooled sensitivity and specificity of 96–98% and 97–98% for RIF resistance, and 95% and 99% for INH resistance, showing equivalence to the Hain GenoType MTBDRplus v2.0 [128,129]. However, as with other LPAs, accuracy decreases in smear-negative samples, which has reinforced WHO’s recommendation to restrict direct use to smear-positive specimens [130].

WHO guidance highlights the assay’s value in tiered diagnostic networks, particularly as a follow-on molecular drug susceptibility test (mDST) in centralized and intermediate laboratories with appropriate biosafety, contamination control, and quality systems [37]. Integration into such networks supports rapid confirmation of MDR-TB, shortening time-to-treatment initiation compared to phenotypic drug susceptibility testing (DST). Per-test costs for NTM + MDRTB II typically range from US$20–30, comparable to Hain LPAs [131].

The Genoscholar™ PZA-TB II assay complements NTM + MDRTB II by specifically targeting pyrazinamide (PZA) resistance. Unlike RIF and INH, resistance to PZA is mediated by diverse mutations scattered throughout the *pncA* coding sequence and its promoter. To address this, the PZA-TB II employs a tiled probe design spanning the full gene, including three probes for synonymous mutations (e.g., Gly60Gly, Ser65Ser, Thr142Thr) that aid in differentiating neutral variants from resistance-conferring changes [132].

WHO recommends PZA-TB II only on culture isolates, reflecting the limited evidence for direct sputum testing and the intrinsic challenges of correlating *pncA* mutations with phenotypic resistance. Accuracy studies report sensitivities of 90–93% and specificities of 90–95% compared with sequencing-informed reference standards, although specificity is affected by the variable phenotypic impact of rare mutations and the limitations of culture-based PZA DST [127,132]. Operationally, the assay is compatible with Nipro’s TwinCubator (12 strips) or MULTIBLOTNS-4800 (48 strips) platforms, with batch runs enabling high-throughput processing. Shelf life is 18 months at 2–10 °C, and costs per strip fall within US$16–30 depending on procurement and volume [131].

Together, the Genoscholar™ NTM + MDRTB II and PZA-TB II LPAs offer a modular approach to molecular resistance profiling. While less flexible than sequencing approaches, they provide faster turnaround than culture DST and can be integrated into diagnostic algorithms alongside Xpert Ultra and next-generation sequencing (NGS) pipelines. Their role is most significant in high-burden countries with centralized laboratories, where early identification of MDR- and PZA-resistant TB informs optimized treatment regimens, improves outcomes, and helps contain transmission [37,99].

### 3.4. Deeplex^®^ Myc-TB

The Deeplex^®^ Myc-TB assay, developed by GenoScreen (Lille, France), is a high-complexity targeted next-generation sequencing (tNGS) platform that enables comprehensive drug-resistance profiling, species identification, and genotyping of *Mycobacterium tuberculosis complex* (MTBC) directly from sputum or cultured isolates. Unlike single-drug assays, Deeplex simultaneously interrogates resistance across 18 MTBC genomic regions, covering first-line drugs (rifampicin, isoniazid, pyrazinamide, ethambutol), fluoroquinolones, aminoglycosides, injectables, and newer agents such as bedaquiline, clofazimine, and linezolid. In addition, it amplifies the *hsp65* gene for species-level identification and the CRISPR locus for spoligotyping, enabling determination of MTBC lineages [133].

The assay is based on a multiplexed amplicon approach: a 24-plex PCR generates targeted fragments, which are prepared for sequencing using Illumina-compatible workflows (e.g., Nextera XT or DNA Flex kits). Sequencing can be run on a variety of Illumina instruments (iSeq, MiniSeq, MiSeq, NextSeq), and analysis is performed via a secure web-based application supported by GenoScreen. The platform provides automated alignment, variant calling, and interpretation based on a continuously curated resistance mutation database [133,134].

WHO endorsed Deeplex Myc-TB in 2023, recognizing it as the first commercial targeted NGS product for TB. It is recommended as a high-complexity molecular drug susceptibility test (mDST) for use in reference laboratories and in patients with bacteriologically confirmed TB requiring extended resistance testing. WHO emphasizes that Deeplex is not a replacement for initial rapid diagnostic tests (WRDs) like Xpert Ultra, but rather a follow-on assay to deliver comprehensive resistance and genotyping data in rifampicin-resistant and multidrug-resistant TB cases [39,80]. Conditional recommendations apply for expanded drug panels, particularly newer agents such as bedaquiline and linezolid, given variable evidence strength [135].

From an analytical standpoint, Deeplex demonstrates strong performance characteristics. It can detect minority resistant subpopulations at proportions as low as 3%, which is particularly important for mixed infections or emerging resistance. The reported limit of detection is 3 CFU/mL in sputum, substantially lower than microscopy and many PCR-based assays [136]. Multicentre clinical evaluations have shown >97% concordance with whole-genome sequencing (WGS) and phenotypic DST across major drug classes [137]. In one prospective implementation study, Deeplex identified MTBC in 79% of direct sputum samples with 100% specificity, correctly detecting all MDR-TB cases [138].

Turnaround time is shorter than WGS pipelines: PCR amplification and library preparation take 4 h, while sequencing and analysis yield results within 48 h, depending on batch size and sequencer throughput. Although longer than cartridge-based NAATs, this timeframe represents a substantial improvement over culture-based DST, which may require weeks [133,136].

Economically, per-test costs are estimated at USD 85 in a 48-sample batch, excluding sequencing equipment and service contracts. While upfront investment is high, scalability and multi-disease sequencing use (e.g., HIV, SARS-CoV-2, antimicrobial resistance surveillance) can improve cost-effectiveness in reference laboratories [80].

In practice, Deeplex Myc-TB occupies a complementary role within diagnostic networks: Xpert or culture establishes initial diagnosis, while Deeplex provides expanded DST and lineage-level insights for treatment guidance and surveillance. Its ability to combine rapid, detailed resistance profiling with epidemiological genotyping positions it as a cornerstone technology for TB programs adopting NGS into routine practice [133,135].

### 3.5. AmPORE-TB^®^

The AmPORE-TB assay, developed by Oxford Nanopore Diagnostics (Oxford, UK) in collaboration with bioMérieux (Marcy-l’Étoile, France), represents a new generation of targeted next-generation sequencing (tNGS) workflows for TB. It simultaneously identifies mycobacterial species, determines MTBC lineages, and detects mutations associated with resistance to first line and second-line anti-TB drugs directly from DNA extracted from decontaminated sputum. Its 27-plex design covers 24 resistance-associated loci for rifampicin, isoniazid, pyrazinamide, ethambutol, fluoroquinolones, aminoglycosides and second-line injectables, ethionamide, bedaquiline, clofazimine, linezolid, delamanid, and pretomanid. In addition, the assay amplifies *hsp65* for MTBC/NTM differentiation and the CRISPR locus for spoligotyping, with an internal process control included [139,140].

Library preparation is based on OND’s Rapid Barcoding Kit, (Oxford Nanopore Diagnostics, Oxford, UK) with sequencing performed on MinION or GridION Q-Line devices (Oxford Nanopore Diagnostics, Oxford, UK). Barcoding permits batching of up to 22–96 samples depending on configuration, and flow cells may be washed and reused to reduce costs. The workflow integrates real-time sequencing with onboard analysis, generating standardized resistance reports aligned to the WHO mutation catalogue [141,142].

WHO’s consolidated TB guidelines (2023) classify AmPORE-TB within high-complexity tNGS and conditionally recommend targeted NGS for drug-resistance detection in bacteriologically confirmed pulmonary TB. Recommended uses include resistance detection for rifampicin, isoniazid, fluoroquinolones, pyrazinamide, and ethambutol, and, in rifampicin-resistant TB, expanded testing for bedaquiline, clofazimine, linezolid, and aminoglycosides [80]. AmPORE-TB is not intended to replace WHO-recommended rapid diagnostic tests (WRDs) but to serve as a follow-on test for prioritized populations requiring rapid, comprehensive molecular DST, such as patients with treatment failure, prior drug exposure, or high likelihood of second-line resistance [39].

Analytical performance demonstrates detection of minority resistant subpopulations down to 10% (heteroresistance threshold), with strong concordance to Deeplex Myc-TB and sequencing-informed reference standards [136,137]. WHO technical reports note class-based performance meeting criteria for rifampicin, isoniazid, fluoroquinolones, linezolid, amikacin, and streptomycin, while results for pyrazinamide and pretomanid require further validation [132]. Importantly, LoD is not expressed in CFU/mL but in coverage metrics, with ≥20× median coverage recommended for reliable variant calling [139].

Operationally, AmPORE-TB enables rapid turnaround. Studies have reported 5–6 h from extracted DNA to final report, including multiplex PCR, library prep, sequencing, and automated analysis [140,143]. This positions the assay for centralized or reference laboratories with sequencing capacity, ISO 13485-compliant quality systems, and bioinformatics support. Batch workflows and barcoding make it scalable, while integration with laboratory information systems allows linkage to national TB reporting networks.

Although currently available as Research Use Only (RUO), AmPORE-TB is progressing through regulatory pathways with distribution and support by bioMérieux. Its deployment within TB programs is expected to complement existing NAATs and phenotypic DST by expanding access to rapid, detailed resistance profiles. WHO guidance emphasizes confirmatory testing for discordant or clinically critical results and careful interpretation of negative calls in low-coverage or low-bacillary samples [39,80].

By combining portability, rapid tNGS workflows, and integration with global mutation catalogues, AmPORE-TB offers a promising tool for modernizing TB diagnostic networks. It strengthens the shift from culture-based DST to comprehensive molecular surveillance while supporting multi-disease sequencing platforms in centralized laboratories [132,139].

### 3.6. TBseq^®^

TBseq^®^ is a high-complexity targeted next-generation sequencing (tNGS) assay developed by Taisho Biomed Instruments (Tokyo, Japan) for comprehensive detection of drug-resistance-associated mutations in *Mycobacterium tuberculosis complex* (MTBC). Designed for use on DNA extracted from culture isolates or, in selected workflows, directly from smear-positive sputum, it employs multiplex PCR amplification of key resistance loci followed by sequencing on Illumina platforms. The assay targets canonical genes implicated in resistance to first line and second-line anti-TB drugs, including *rpoB*, *katG*, *inhA* promoter, *pncA*, *embB*, *gyrA*, *gyrB*, *rrs*, and *eis* promoter, while also covering loci linked to resistance against bedaquiline, clofazimine, linezolid, delamanid, and pretomanid [144,145].

The workflow begins with DNA extraction from heat-inactivated isolates or decontaminated sputum, followed by multiplex PCR amplification and Illumina-compatible library preparation. Sequencing can be performed on benchtop instruments such as MiSeq or MiniSeq, with optimized read depths enabling detection of minority variants at frequencies as low as 5–10% [146]. Bioinformatic analysis is performed by the proprietary TBseq^®^ pipeline, which aligns reads to the *M. tuberculosis* H37Rv reference genome, calls variants, and annotates them using curated drug-resistance databases consistent with the WHO mutation catalogue [142,147].

WHO’s latest guidelines classify TBseq^®^ within the high-complexity targeted NGS group, recommended for centralized or reference laboratories with advanced molecular capacity, quality assurance systems, and bioinformatics expertise [80]. It is not intended as a frontline diagnostic test but rather for patients requiring detailed drug susceptibility testing (DST), particularly those with rifampicin-resistant or multidrug-resistant TB where regimen optimization is critical. Its use is prioritized in high-risk groups, such as individuals with prior TB treatment or exposure to new/repurposed drugs [39].

**Figure 2:**
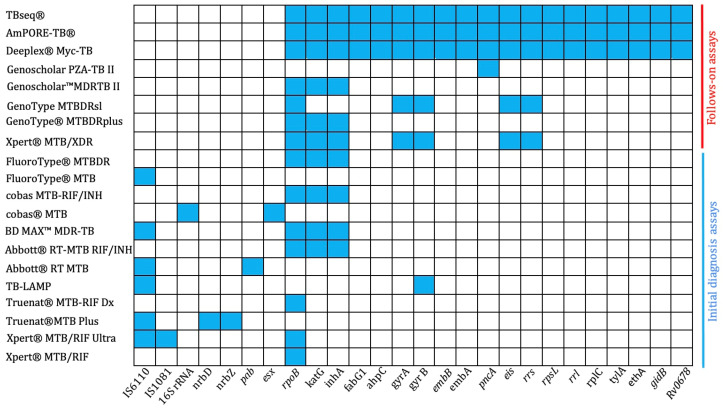
Heatmap of genetic target coverage in WHO-endorsed and commercial molecular assays for *Mycobacterium tuberculosis* diagnosis and drug resistance detection: This heatmap depicts the genetic targets detected by a range of WHO-endorsed and commercial molecular assays for MTB diagnosis and drug resistance determination. Rows represent individual assays, with the blue bracket on the right indicating initial diagnosis assays designed for primary TB detection with or without limited resistance detection, and the red bracket indicating follow-on assays primarily intended for drug resistance detection following an initial TB diagnosis. Columns correspond to specific genetic targets or markers. Blue squares indicate that the assay detects mutations in the corresponding gene or locus, while white squares indicate that the assay does not include that target in its detection panel.

A comparative synthesis of follow-on assays for the detection of drug resistance, with details on technical specifications, recommendations, advantages, and constraints, is provided in Table 2. The breadth of genetic target coverage across these assays is further depicted in Figure 2.

## 4. Structural and Financial Constraints Limiting NAAT Scale-Up

Despite their transformative contribution to TB diagnostics, WHO-endorsed NAATs continue to face substantial limitations that restrict their equitable deployment, particularly in low- and middle-income countries (LMICs). Most currently implemented cartridge-based or moderate- to high-complexity NAAT platforms rely on stable electricity, climate-controlled laboratory environments, uninterrupted supply chains for proprietary consumables, and regular preventive maintenance. These infrastructural prerequisites remain difficult to ensure in decentralized or peripheral health facilities, where power instability, limited cold-chain capacity, and weak logistics systems are common challenges [22,80,148,149]. Implementation studies of Xpert MTB/RIF and Xpert Ultra consistently demonstrate that the physical placement of instruments alone does not guarantee diagnostic impact. Instead, recurrent operational constraints, including cartridge stock-outs, equipment downtime, delayed maintenance, insufficient biosafety infrastructure, and fragile specimen-referral networks, frequently undermine test utilization and delay clinical decision-making [148,150,151]. These challenges are further exacerbated in settings with low testing volumes, where fixed operational costs translate into higher per-test expenses, undermining cost-effectiveness and long-term sustainability [9,152,153,154,155,156,157,158]. As a result, many national TB programs remain dependent on external donor funding to maintain NAAT access, raising concerns regarding financial resilience and scalability.

### 4.1. Operational and Diagnostic Performance Gaps of NAAT Platforms

Beyond infrastructure and cost constraints, several intrinsic technical and operational limitations persist across existing NAAT platforms. Most assays demonstrate reduced sensitivity in paucibacillary disease, including pediatric TB, extrapulmonary TB, and TB in people living with HIV. These populations, which already experience disproportionate diagnostic delays, continue to face substantial gaps in microbiological confirmation [9,155].

On the other hand, biosafety considerations further complicate NAAT implementation. The reliance on raw sputum as the primary specimen exposes healthcare workers and nearby patients to aerosolized *Mycobacterium tuberculosis*, a risk amplified in crowded clinics with inadequate ventilation or insufficient respiratory protection [153,154]. In many LMIC facilities, biosafety cabinets and appropriate infection-control measures remain limited, constraining safe specimen handling and molecular processing.

Otherwise, dependence on expectorated sputum also poses major constraints for pediatric TB diagnosis. Children frequently present with paucibacillary disease and are often unable to produce sputum, rendering many molecular assays less applicable [6,159]. Alternative respiratory specimens such as induced sputum or gastric aspirates are invasive, logistically demanding, and require trained personnel and specialized infrastructure, with suboptimal diagnostic yield even when available [38,160,161,162].

### 4.2. Human Resource Capacity and Biosafety Considerations

The effective use of NAATs requires personnel trained in molecular workflows, instrument troubleshooting, biosafety practices, and laboratory quality management systems. However, such expertise is often limited in high-burden LMIC settings, particularly at peripheral or district-level facilities [152]. High staff turnover, insufficient continuing education, and competing clinical workloads further undermine sustained capacity building.

Quality assurance remains a critical challenge. External quality assessment (EQA) schemes for molecular diagnostics are inconsistently implemented across endemic regions, increasing the risk of undetected performance drift, contamination, or operator-related errors. Regulatory oversight of NAAT platforms also varies widely between countries, leading to delays in national registration, restricted procurement, and slow adoption of updated assay generations. Streamlining regulatory pathways while maintaining rigorous performance validation remains essential for accelerating equitable NAAT access.

### 4.3. Toward Decentralized, Non-Conventional Specimen NAATs

Collectively, these financial, infrastructural, biosafety, and workforce limitations highlight a persistent disconnect between WHO policy recommendations and real-world NAAT implementation capacity. Addressing this gap will require the development of molecular diagnostics that are simpler, more affordable, less infrastructure-dependent, and adaptable to decentralized care settings [22,149].

Encouragingly, NAAT-based testing on alternative, non-conventional specimen types is beginning to mitigate some of these barriers. Stool-based Xpert MTB/RIF and Xpert Ultra assays have demonstrated clinically meaningful sensitivity for pediatric TB, leading to WHO endorsement of stool Xpert as a molecular alternative when respiratory specimens cannot be obtained [163,164,165]. These approaches preserve NAAT analytical rigor while expanding diagnostic accessibility in vulnerable populations [166,167,168,169,170].

In parallel, other molecular innovations are emerging to reduce reliance on sputum and centralized laboratories. Oral-swab and nasopharyngeal PCR-based assays are under evaluation as minimally invasive NAAT strategies for community-based screening and pediatric testing [171]. Building further on this trajectory, a rapidly expanding pipeline of next-generation molecular platforms aims to overcome key limitations of current NAATs, including turnaround time, infrastructure dependence, and sensitivity in low-bacillary-load disease.

Among the most promising developments are CRISPR-based NAAT systems, which leverage programmable nucleic acid recognition coupled with isothermal amplification and collateral cleavage-based signal generation. These assays offer ultra-high analytical sensitivity, rapid time to result, and the potential for simplified workflows compatible with near-point-of-care deployment. Collectively, such innovations represent a fundamental evolution of NAAT technology and may play a central role in extending molecular TB diagnostics beyond centralized laboratories toward truly decentralized, high-impact testing strategies.

### 4.4. Emerging CRISPR-Based NAAT Tools for Rapid Tuberculosis Diagnosis

Recent advances in CRISPR-based molecular technologies have introduced a new class of emerging tools with potential relevance for tuberculosis (TB) diagnosis. These assays aim to address several limitations of conventional nucleic acid amplification tests (NAATs), including infrastructure dependence, turnaround time, and reduced sensitivity in low-bacillary-load disease. By combining programmable nucleic acid recognition with isothermal amplification and signal amplification via collateral cleavage, CRISPR-based platforms represent a distinct methodological evolution within the NAAT landscape.

Among the most extensively studied systems is DETECTR (DNA Endonuclease-Targeted CRISPR Trans Reporter), which utilizes the collateral cleavage activity of Cas12a following sequence-specific recognition of Mycobacterium tuberculosis genomic targets. Activation of Cas12a results in nonspecific cleavage of labeled reporter molecules, generating either fluorescent or lateral-flow readouts under isothermal conditions [172]. The elimination of thermocycling and the reduction in instrumentation requirements have been proposed as advantages for decentralized testing contexts, although most evaluations to date remain at the proof-of-concept or early validation stage.

Similarly, the SHERLOCK platform (Specific High-sensitivity Enzymatic Reporter unLOCKing) employs Cas13a-mediated RNA-guided collateral cleavage to achieve very high analytical sensitivity, with reported limits of detection extending into the attomolar range [172]. A notable feature of SHERLOCK is its modular design, whereby CRISPR RNA guides can be reprogrammed to detect specific genomic regions or resistance-associated mutations, including those linked to rifampicin, isoniazid, and fluoroquinolone resistance [173]. This flexibility suggests potential applicability in the detection of drug-resistant TB; however, further clinical validation is required to determine performance across diverse epidemiological and operational settings.

Building on these foundational platforms, several emerging CRISPR-based TB assays are integrating sample preparation, isothermal target amplification, most commonly through recombinase polymerase amplification or loop-mediated isothermal amplification, and CRISPR-mediated detection into streamlined workflows with total assay times of less than one hour [174]. Experimental studies have also explored the use of freeze-dried CRISPR reagents, enabling ambient-temperature storage and transport, which could reduce logistical constraints in resource-limited environments [175,176]. In parallel, prototype instrument-free formats, including paper-based lateral-flow strips and smartphone-assisted fluorescence detection systems, are under development and may further support decentralized or community-level testing strategies [177].

Taken together, CRISPR-based assays represent a rapidly evolving category of emerging NAAT tools for TB diagnosis. While their analytical performance and operational characteristics are promising, most platforms remain at an early stage of development, and robust clinical validation, cost-effectiveness analyses, and implementation studies will be required before their potential role alongside or beyond existing WHO-endorsed NAATs can be clearly defined.

### 4.5. Gaps in the Implementation of Upfront Molecular Testing

Despite robust evidence supporting molecular diagnostics and clear WHO recommendations advocating NAATs as initial diagnostic tools for tuberculosis, the implementation of upfront molecular testing has remained heterogeneous across settings and over time. Historically, many high-burden countries relied on smear microscopy as the first-line diagnostic approach, with molecular assays introduced only as confirmatory or second-line tests, resulting in delayed detection of both tuberculosis and drug resistance) [3,80]. Although WHO has progressively recommended molecular tests as the preferred initial diagnostic method since 2010, the transition from microscopy-based to molecular-first algorithms has been uneven and often protracted, particularly in resource-limited and decentralized settings [10,37].

Multiple factors have contributed to this slow and inconsistent adoption, including financial constraints, limited availability and placement of molecular platforms, infrastructure and maintenance requirements, supply chain challenges, and the need for sustained training of laboratory personnel [80,128]. In addition, health system inertia and the coexistence of legacy diagnostic algorithms have frequently resulted in parallel testing strategies, where molecular assays are reserved for selected patient groups rather than implemented universally as upfront tests [24]. WHO programmatic assessments and implementation studies have repeatedly highlighted gaps between policy recommendations and real-world diagnostic practices, particularly during the early and intermediate phases of molecular test scale-up [12,36].

These persistent gaps may substantially compromise the anticipated public health impact of molecular diagnostics, including delayed treatment initiation, missed or late detection of drug resistance, and continued transmission at the community level [2,88]. Addressing these implementation challenges remains critical to fully realizing the benefits of upfront molecular testing and achieving the goals of timely, accurate, and patient-centered tuberculosis diagnosis.

### 4.6. Programmatic Advantages of Upfront NAAT and Reuse of Molecular Material for Reflex Testing

Beyond its role in initial case detection, upfront molecular testing offers substantial programmatic and operational advantages that extend across the entire diagnostic cascade. When NAATs are used as first-line assays, the extracted mycobacterial DNA can be retained and repurposed for downstream molecular analyses, including line probe assays (LPAs) and targeted sequencing approaches, without the need for additional patient specimens [12]. This reflex testing strategy is particularly valuable in high-burden and resource-limited settings, where repeated sample collection is often associated with delays, increased laboratory workload, and a heightened risk of patient loss to follow-up. By leveraging NAAT-derived DNA, diagnostic pathways can be streamlined, enabling more rapid confirmation of drug resistance and earlier initiation of appropriate treatment regimens.

Moreover, the reuse of molecular material supports a more integrated and patient-centered diagnostic workflow by reducing diagnostic turnaround times and minimizing dependence on culture-based methods, which are time-consuming and require advanced biosafety infrastructure. Programmatic studies have demonstrated that such molecular-first algorithms facilitate continuity of care by allowing resistance profiling to be performed immediately after TB detection, thereby shortening the interval between diagnosis and optimized therapy [70]. This approach also enhances laboratory efficiency by optimizing specimen utilization and reducing redundant testing steps, particularly in decentralized laboratories where specimen transport and storage remain logistical challenges.

From a public health perspective, upfront NAAT combined with reflex molecular testing contributes to improved surveillance of drug-resistant tuberculosis by enabling earlier and more comprehensive resistance characterization at the population level. The ability to rapidly escalate from initial detection to resistance testing using the same molecular material strengthens the responsiveness of TB control programs and aligns with WHO recommendations promoting integrated molecular diagnostic strategies [10]. Collectively, these operational, clinical, and surveillance advantages reinforce the central role of upfront molecular testing not only as a diagnostic tool, but as a foundational component of modern tuberculosis diagnostic and care pathways.

## 5. Conclusions

Over the last decade, NAATs have profoundly transformed TB diagnosis by reliance on slow, culture-based methods. Cartridge-based systems now provide rapid, decentralized testing, while more advanced tools such as LPAs and targeted sequencing assays offer comprehensive drug resistance profiling at reference laboratories. Together, these approaches enable faster initiation of effective regimens and strengthen surveillance of emerging resistance. Nevertheless, important challenges remain. Limited affordability, infrastructure requirements, and uneven uptake across high-burden settings continue to restrict the global impact of these technologies. Moreover, no single assay currently addresses all clinical and programmatic needs, from pediatric and extrapulmonary TB to the detection of resistance beyond first- and second-line drugs. The way forward will require integrating NAATs into diagnostic networks that combine point-of-care accessibility with centralized sequencing capacity, while ensuring sustainability through equitable pricing and programmatic support. By bridging technological innovation with health system realities, molecular diagnostics can help close the persistent diagnostic gap and contribute to global TB elimination effort.

## Figures and Tables

**Table 1:** First-line molecular assays for the diagnosis of MTB.

	First-Line Diagnostic Tools for TB Diagnosis											
	Low-Complexity NAATS					Moderate-Complexity NAATS						
	Xpert^®^ MTB/RIF	Xpert^®^ MTB/RIF Ultra	Truenat^TM^ MTB/MTB Plus	Truenat^TM^ MTB RIF DX	TB-LAMP	ABBOT RealTime MTB	ABBOT RT MTB-RIF/NIH	BD MAX^TM^ MDR TB	cobas^®^ MTB	cobas^®^ MTB-RIF/NIH	FluoroType^®^ MTB	FluoroType^®^ MTBDR
Company	Cepheid	Cepheid	Molbio Diagnostic	Molbio Diagnostic	Eiken Chemical Co., Ltd.	Abbot	Abbot	Becton Dickinson	Roche Diagnostics	Roche Diagnostics	Bruker-Hain Lifescience	Bruker-Hain Lifescience
Year *	2010	2017	2020		2012	2014	2017	2018	2008	2008	2016	2018
Country	USA	USA	India	India	Japan	USA	USA	USA	Switzerland	Switzerland	Germany	Germany
Technology	Automated RT-PCR (molecular beacons)	Automated RT-PCR (molecular beacons)	RT micro-PCR on chip	RT micro-PCR on chip	LAMP	Automated RT-PCR (m2000 system)	Automated RT-PCR (m2000 system)	Automated RT-PCR	RT-PCR	RT-PCR	RT-PCR (melting curve)	RT-PCR (melting curve)
Detects	MTBC + resistance to RIF	MTBC + resistance to RIF	MTBC	MTB + resistance to RIF	MTB	MTBC	MTB + RIF resistance	MTBC + RIF & INH resistance	MTB	MTB DNA	MTB	MTB + RIF & INH resistance
Target (gene)	*rpoB*	*rpoB*, *IS6110*, *IS1081*	*nrdB*, *nrdZ*, *IS6110*	*rpoB*	*IS6110*, *gyrB*	*IS6110*, *paB*	*rpoB*, *katG*, *inhA*	*IS6110*, *rpoB*, *katG*, *inhA*	*16S rRNA*, *esx genes*	*rpoB*, *katG*, *inhA*	*IS6110*	*rpoB*, *katG*, *inhA*
Limit of Detection (CFU/mL)	131 (TB) 112 (RIF)	Between 15–150	100 (Truenat MTB); 30 (Truenat MTB+)	100	10	17 (TB), 60 (RIF/INH)	60	0.5 (TB), 6 (RIF/INH)	7.6	12.6	15	14
Time to Detection	2 h	1.5 h	1 h	1 h	1–1.5 h	10.5 h	10.5 h	4 h	3.5 h	3.5 h	3 h	3 h
WHO-endorsed	Yes	Yes	Yes	Yes	Yes	Yes	Yes	Yes	Yes	Yes	Yes	Yes
Recommendations	❶	❶	❷	❷	❸	❸	❹	❺	❺	❹	❺	❹
	First-Line Diagnostic Tools for TB diagnosis											
	Low-complexity NAATS					Moderate-complexity NAATS						
Test specification	Xpert^®^ MTB/RIF	Xpert^®^ MTB/RIF Ultra	Truenat^TM^ MTB/MTB plus	Truenat^TM^ MTB RIF DX	TB-LAMP	ABBOT RealTime MTB	ABBOT RealTime MTB-RIF/NIH	BD MAX^TM^ MDR TB	cobas^®^ MTB	cobas^®^ MTB-RIF/NIH	FluoroType^®^ MTB	FluoroType^®^ MTBDR
Benefits	• Fully automated cartridge NAAT • Detects TB + RIF resistance in ~2 h • Low biosafety requirements • Minimal hands-on time	• Higher sensitivity than MTB/RIF, esp. in smear− & pediatric cases • Same workflow & runtime as MTB/RIF • Lower limit of detection (~16 CFU/mL) • Semi-quantitative results	• Portable, battery-operated platform • Works in primary healthcare/POC settings • Detects TB in ~1 h • Minimal biosafety requirements	• Companion reflex test for RIF resistance • Uses same small-footprint Truelab platform • Rapid (<1 h) • Suitable for POC use in RLS	* Rapid turnaround * Simple readout * WHO-endorsed as a replacement for smear microscopy	• High-throughput PCR platform (m2000) • Detects TB from various sample types • Automated extraction and amplification • High specificity	• Detects RIF & INH resistance • Automated, high-throughput on m2000 system • Good performance from smear+ samples • Integrates with Abbott MTB assay	• Automated sample-to-result PCR system • Detects TB, RIF & INH resistance • Closed-cartridge format reduces contamination risk • Random-access testing	• Automated sample prep + PCR on cobas 6800/8800 • High-throughput for TB detection • Works with multiple sample types • Low hands-on time	• Companion assay for cobas MTB • Detects RIF & INH resistance • Fully automated workflow • High-throughput	• PCR-based with automated detection (FluoroCycler) • High throughput & minimal contamination risk • Can process multiple samples in parallel • Suitable for central labs	• Detects TB + RIF & INH resistance • Automated PCR with melt curve analysis • Integrates with FluoroCycler system • Faster than manual LPAs
Limitations	• Only detects RIF resistance (*rpoB*) • Misses INH and other drug resistance • Limited to mutations within 81-bp *rpoB* core • Cartridge cost and infrastructure needs	• Still only RIF resistance detection • Slightly lower specificity in some low-prevalence settings • Cannot detect minority variants • Cartridge cost	• Does not detect drug resistance (needs reflex test) • Requires Truenat MTB RIF Dx for DST • Limited throughput • Consumable availability outside India can be limited	• Only detects RIF resistance • Limited to *rpoB* hotspot mutations • Lower throughput than lab-based systems • Requires initial Truenat MTB positive result	•Detects TB only • Lower sensitivity compared with Xpert MTB/RIF and Ultra •Manual DNA extraction required • Limited uptake and availability	• No drug resistance detection (requires MTB RIF/INH kit) • Large lab-based equipment • Higher infrastructure requirements • Not suitable for POC	• Requires large automated platform • Limited to *rpoB, katG, inhA* • Not POC-suitable • Limited access outside high-resource labs	• Requires BD MAX instrument • Limited to *rpoB, katG, inhA* • Not widely available in all regions • Higher per-test cost in low-volume settings	• No drug resistance detection (needs cobas MTB RIF/INH) • Large, high-cost instruments • Requires central lab infrastructure • Not portable	• Requires cobas platform • Limited to *rpoB, katG, inhA* • Not POC-suitable • Limited field validation in LMICs	• TB detection only (no DST) • Requires proprietary instrument • Not portable • Limited availability outside certain regions	• Requires specific platform • Limited to *rpoB, katG, inhA* mutations • Not suitable for POC • Limited global availability
References	[1,5,6,9,10,11,12,13,19,20,21,22,23]	[11,25,30,31,32,34,35,41]	[14,40,41,45,47,48,50]	[14,40,41,43,44,46,48,50]	[37,51,53,54,55,56,57,58,59,60,61,62]	[37,63,64,65,66,67,68,69,70,71,72,73,74,75,76,77,78]	[63,64,65,69,70,71,73,75,76]	[37,79,80,81,82,83,84,85,86]	[39,99,100,101,102,103,104,108,110]	[39,99,100,101,102,103,104,105,106,107,108,109,110]	[37,93,94,95,96]	[94,96,97,99]

(*): Year of the assay WHO endorsement, h: hours, RIF: Rifampicin, INH: Isoniazid, MTB: *Mycobacterium tuberculosis*, MTBC: *Mycobacterium tuberculosis* complex, DST: Drug Susceptibility Testing, LPA: Line Probe Assay, CFU: Colony-Forming Unit, RT-PCR: Real Time Polymerase Chain Reaction, LAMP: Loop-Mediated Isothermal Amplification, POC: Point Of Care, RLS: resource-limited settings, LMIC: Low and Middle Income Countries, NAAT: Nucleic acid amplification testing. ❶ Detection of TB & RIF resistance in sputum from patients with signs and symptoms of pulmonary TB. ❷ Detection of TB in sputum from with signs and symptoms of PTB. ❸ Detection of PTB from individuals with signs and symptoms of PTB. ❹ Detection of RIF & INH resistance in respiratory samples from individuals with signs and symptoms of PTB. ❺ Detection of PTB in respiratory samples from individuals with signs and symptoms of PTB.

**Table 2:** Follow-on assays for the detection of TB and drugs resistance.

	Follow-On Assays for TB Drug Resistance Profiling							
	Low Complexity	Moderate Complexity				High Complexity		
Test specification	Xpert^®^ MTB/XDR	GenoType^®^ MTBDRplus	GenoType^®^ MTBDRsl	Genoscholar ^TM^ NTM + MDRTB II	Genoscholar ^TM^ PZA-TB II	Deeplex^®^-MycTB	AmPORE-TB^®^	Tbseq^®^
Company	Cepheid	Bruker-Hain Lifescience	Bruker-Hain Lifescience	Nipro	Nipro	Genoscreen/Illunmina	Nanopore Oxford Technologies	Shengting Medical Technology
Year	2021	2008	2012	2011	2011	2018	2024	2024
Country	USA	Germany	Germany	Japan	Japan	France	UK	China
Technology	Automated RT-PCR	Multiplex PCR + LiPA	Multiplex PCR + LiPA	Multiplex PCR + LiPA	Multiplex PCR + LiPA	Targeted NGS	Targeted NGS	Targeted NGS
Detects	MTB + resistance to INH, FLQ, SLIDs	MTB + resistance to RIF and INH	MTB + resistance to FLQ and SLIDs	MTBC + resistance to RIF and INH	MTBC + resistance to Pyrazinamide	MTB and resistance mutations	MTB and resistance mutations	MTB and resistance mutations
Target	*rpoB, katG, inhA, oxyR-aphC, fabG1, gyrA, gyrB, eis, rrs*	*rpoB, katG, inhA*	*gyrA, gyro, rrs, eis*	*rpoB, katG, inhA*	*pncA*	24 resistance genes + genotyping	24 resistance genes + genotyping	24 resistance genes + genotyping
Limit of detection	71.9 CFU/mL	10,000 CFU/mL	10,000 CFU/mL	10,000 CFU/mL	10,000 CFU/mL	100 CFU/mL	11.5 CFU/mL	Not available
Time to Detection	1.5 h	5 h	5 h	5 h	5 h	48 h	5 h	12 h
Who-endorsed	Yes	Yes	Yes	Yes	Yes	Yes	Yes	Yes
Recommendations	Detection of resistance to INH and FLQ in sputum from people with bacteriologically confirmed PTB; and detection of AMK and ETH resistance in those with RIF resistance	Detection of resistance to RIF and INH in smear-positive sputum or cultured isolates from people with PTB	Detection of resistance to FLQ and AMK in sputum or cultured isolates from patients with confirmed MDR/RR-TB	Detection of resistance to RIF and INH in smear-positive sputum or cultured isolates from people with PTB	Detection of PZA resistance in cultured isolates from people with PTB	Detection of resistance to first- and selected SLIDs in respiratory samples.	Detection of resistance to RIF, INH, FLQ, Lzd, AMK and SM in respiratory samples from people with bacteriologically confirmed PTB	Detection of EMB resistance in respiratory samples from people with bacteriologically confirmed PTB
Benefits	• Fully automated cartridge system • Detects INH (with two additional targets), FLQ, AMK, KAN, CAP, ETH in one test • Low biosafety requirements	• Detects RIF & INH resistance directly from smear+ sputum or culture • Covers *rpoB, katG, inhA* promoter • 1-day turnaround • Lower cost than NGS	• Detects FLQ, SLIDs, ETH resistance • Targets *gyrA/B, rrs, eis, ethA* • WHO-endorsed follow-on test • 1-day turnaround	• Detects MTBC vs. NTM + RIF & INH resistance • Strip LPA usable in moderate labs • Common mutations in *rpoB, katG, inhA* • ~5-h turnaround	• Targets *pncA* & upstream promoter • Rapid alternative to phenotypic PZA DST • Wide mutation coverage across *pncA* • Compatible with LPA workflows	• 24 resistance genes + lineage typing • Illumina sequencing for high accuracy • Clinical + surveillance use	• Nanopore targeted sequencing • Portable & potentially field-deployable • Broad DST + lineage info • Minority variant detection	• Broad targeted NGS panel • Rare/novel/mixed variant detection • Lineage + phylogenetics • Surveillance & outbreak utility
**Limitations**	• High cartridge cost • Limited mutation coverage • Requires stable electricity/temp control • Limited mutation coverage • Cannot detect minority variants	• Manual extraction & hybridization • Misses rare mutations • Requires moderate-complexity lab • Reduced sensitivity in smear−	• Limited mutation spectrum • Needs high bacterial load or culture • Moderate lab infrastructure needed • No minority variant detection	• Limited to RIF & INH • Manual hybridization • Lower sensitivity smear− • Misses novel variants	• Requires DNA extraction & hybridization • May miss rare mutations outside target • Needs moderate lab setup • Limited global availability	• Needs high-complexity sequencing lab • Slower than cartridge tests • High per-sample cost unless batched • Not POC-suitable	• Specialized library prep needed • Requires bioinformatics capacity • Lower accuracy than Illumina if QC poor • Limited implementation data	• Needs high-throughput sequencing • Not rapid POC • High cost; batching needed • Longer turnaround than NAATs
**References**	[39,99,112,113,114,115,116,117,118,119]	[9,39,92,99,120,121,122,123,124,125]	[9,39,92,99,120,121,122,123,124,125]	[37,39,99,126,127,128,129,130,131,132]	[37,39,99,126,127,128,129,130,131,132]	[39,80,132,133,134,135,136,137,138]	[39,80,132,136,137,139,140,141,142,143]	[39,80,142,144,145,146,147]

h: hours, CFU: Colony-Forming Unit, RIF: Rifampicin, INH: Isoniazid, MTB: *Mycobacterium tuberculosis*, MTBC: *Mycobacterium tuberculosis* complex, PZA: Pyrazinamide, DST: Drug Susceptibility Testing, LPA: Line Probe Assay, FLQ: Fluoroquinolones, SLIDs: Second-Line Injectable Drugs, EMB: Ethambutol, AMK: Amikacin, CFU: Colony-Forming Unit, RT-PCR: Real Time Polymerase Chain Reaction, POC: Point Of Care, LMIC: Low and Middle Income Countries, Lzd: Linezolid, SM: streptomycin, PZA: pyrazinamide, PTB: pulmonary tuberculosis, ETH: Ethionamide, LiPA: reverse hybridization line probe assay, RT-PCR: Real-time Polymerase Chain Reaction, NGS: Next-Generation Sequencing, MDR/RR-TB: Multidrug- and rifampicin-resistant tuberculosis, NTM: Non-tuberculous mycobacteria.

## Data Availability

No new data were created or analyzed in this study. Data sharing is not applicable to this article.

## Abbreviations

The following abbreviations are used in this manuscript:

AMK	Amikacin
BDQ	Bedaquiline
CFU	Colony-Forming Unit
CFZ	Clofazimine
DST	Drug Susceptibility Testing
EMB	Ethambutol
FLQ	Fluoroquinolones
INH	Isoniazid
LAMP	Loop-Mediated Isothermal Amplification
LPA	Line Probe Assay
LZD	Linezolid
MDR-TB	Multidrug-Resistant Tuberculosis
MTB	*Mycobacterium tuberculosis*
MTBC	*Mycobacterium tuberculosis* complex
NGS	Next-Generation Sequencing
RT-PCR	Real-Time Polymerase Chain Reaction
PZA	Pyrazinamide
RIF	Rifampicin
TB	Tuberculosis
SLID	Second-Line Injectable Drugs
WHO	World Health Organization
XDR-TB	Extensively Drug-Resistant Tuberculosis
